# Surface Survival and Internalization of *Salmonella* through Natural Cracks on Developing Cantaloupe Fruits, Alone or in the Presence of the Melon Wilt Pathogen *Erwinia tracheiphila*

**DOI:** 10.1371/journal.pone.0105248

**Published:** 2014-08-22

**Authors:** Dhiraj Gautam, Shefali Dobhal, Mark E. Payton, Jacqueline Fletcher, Li Maria Ma

**Affiliations:** 1 National Institute for Microbial Forensics & Food and Agricultural Biosecurity, Department of Entomology and Plant Pathology, Oklahoma State University, Stillwater, Oklahoma, United States of America; 2 Department of Statistics, Oklahoma State University, Stillwater, Oklahoma, United States of America; University of Osnabrueck, Germany

## Abstract

Outbreaks of foodborne illness attributed to the consumption of *Salmonella*-tainted cantaloupe have occurred repeatedly, but understanding of the ecology of *Salmonella* on cantaloupe fruit surfaces is limited. We investigated the interactions between *Salmonella enterica* Poona, the plant pathogenic bacterium *Erwinia tracheiphila*, and cantaloupe fruit. Fruit surfaces were inoculated at the natural cracking stage by spreading *S. enterica* and *E. tracheiphila*, 20 µl at 10^7^ cfu/ml, independently or together, over a 2×2 cm rind area containing a crack. Microbial and microscopic analyses were performed at 0, 9 and 24 days post inoculation (DPI). Even at 24 DPI (fruit maturity) *S. enterica* was detected on 14% and 40% of the fruit inoculated with *S. enterica* alone and the two-pathogen mixture, respectively. However, the population of *S. enterica* declined gradually after initial inoculation. *E. tracheiphila*, inoculated alone or together with *Salmonella*, caused watersoaked lesions on cantaloupe fruit; but we could not conclude in this study that *S. enterica* survival on the fruit surface was enhanced by the presence of those lesions. Of fruit inoculated with *E. tracheiphila* alone and sampled at 24 DPI, 61% had watersoaked lesions on the surface. In nearly half of those symptomatic fruits the watersoaking extended into the sub-rind mesocarp, and *E. tracheiphila* was recovered from that tissue in 50% of the symptomatic fruit. In this work, *E. tracheiphila* internalized through natural cracks on developing fruits. *S. enterica* was never detected in the fruit interior (ca. 2–3 mm below rind surface) under the limited conditions of our experiments, but the possibility that it, or other human pathogens that contaminate fresh produce, might also do so should be investigated under a wider range of conditions and produce types.

## Introduction

Incidences of the contamination of fresh fruits and vegetables with human pathogens and resulting foodborne illness outbreaks have been increasing in the United States and around the world [1]–[3]. *Salmonella enterica*, the causal agent of salmonellosis, is one of the most common human pathogenic bacteria contaminating fresh produce world-wide [2],[4]. Among recent *Salmonella*-associated disease outbreaks, cantaloupe (*Cucumis melo* var. *reticulatus*) was one of the most implicated produce types [5]–[10]. The first documented salmonellosis outbreak, caused by consumption of salad bar cantaloupes contaminated with *S. enterica* Chester in 1990, involved 245 reported cases in 30 U.S. states [11]. Cantaloupe fruits have pronounced rind netting containing sheltered niches likely to harbor microorganisms and hamper effective sanitation [12]–[15]. As few as 150 bacteria cm^−2^ on the rind surface can contaminate the edible mesocarp upon slicing [14].

The formation of netting on cantaloupe fruit begins at the blossom end, when the fruits are 10–12 days old, with natural cracking of the rind [16]. The cracks lengthen and cover the entire fruit surface by the end of the fruit-expansion stage [16]. Stomata on the fruit surface become nonfunctional over time. Then, a thick cuticle containing gas-exchanging lenticels forms, sealing the cracks [16].

As the rind cracks form, defensive compounds are produced by the plant to reinforce structural and chemical barriers against microbial attack [16]. Cantaloupe fruits usually develop on the soil surface where the physical defensive barriers may be compromised, providing a route of entry for saprophytes or plant/human pathogens present in the agricultural environment [17]. Fruit rot is a very common problem in cantaloupes that develop on soil surfaces (James Motes, Oklahoma State University, Department of Horticulture, retired; personal communication).

Human pathogens such as *S. enterica* can be brought into agricultural fields by contaminated irrigation water [18]–[20], rain splash [21],[22], insect vectors [23], or soil and crop debris [24],[25], and contaminate the growing plants [17],[21],[22],[25]. Bacterial uptake and translocation by and within plant parts following artificial inoculation have been reported in many plant species [24],[26]–[30]. Although the ecology of *S. enterica* on plant surfaces is poorly understood, several researchers have shown that the presence of other plant resident microorganisms, such as soft rotting or foliar leaf-spotting bacteria [31],[32] or fungi [33]–[35], can promote the growth and colonization of plants by *S. enterica*. The presence of other microorganisms or wounds on fruit or leaf surfaces can influence the survival of human pathogens on those surfaces. Barak and Liang [24] and Potnis et al. [32] reported significantly higher *S. enterica* populations on tomato leaves after the human pathogen was inoculated together with *Xanthomonas* species than when it was inoculated alone. Similar synergism was reported between species of *Rhizopus* or *Botrytis*, both of which cause rots in vegetables, and *S. enterica* Typhimurium [35]. Brandl et al. [36] showed synergism in attachment and biofilm formation between *S. enterica* and *Aspergillus niger*, possibly due to cellulose-chitin interaction. *S. enterica* failed to form biofilms when it was either pre-incubated with N- acetylglucosamine (a monomeric component of chitin) or when a cellulose-deficient mutant was used. Similarly, co-inoculation of *S. enterica* with *Cladosporium cladosporioides* significantly enhanced the ability of the former to penetrate into the mesocarp of cantaloupe fruit during post harvest storage [33].

Whether plant or human pathogens can internalize through openings created on the cantaloupe rind surface at the time of cracking is not known, and the possible interactions between plant and human pathogens in this niche have not been explored. The objective of this study was to investigate the survival, and the possibility of internalization, of the human pathogen *S. enterica*, inoculated onto cantaloupe fruits at the time of natural rind cracking, and whether the presence of the plant pathogen *Erwinia tracheiphila* (cause of cucurbit bacterial wilt) influenced the fitness of the human pathogen. The results of this work will help to identify strategies to limit contamination and internalization by human pathogens on this popular and nutritious fruit.

## Materials and Methods

### Bacterial strains, labeling, storage and inoculum preparation

A clinical isolate of *S. enterica* Poona from a 2001 disease outbreak in the United States, linked to cantaloupes imported from Mexico, was labeled in our laboratory with pUC18T-mini-Tn7T-Gm-dsRedExpress (fluorescing red) having gentamycin and ampicillin resistance genes, following the protocol of Choi and Schweizer [37]. *E. tracheiphila* strain MCM1-1, isolated from Oklahoma cantaloupe by B. Bruton (USDA-ARS, Lane, OK) and provided by M. Gleason (Iowa State University, IA) was transformed with pGFPuv (Clontech Laboratories, Inc., CA) by electroporation as described by Ma et al. [38] and colonies were selected after growing on ampicillin amended nutrient agar. Plasmid stability tests were performed for both labeled pathogens followed by plating on nutrient agar (NA; BBL, Becton Dickinson, Sparks, MD) or Luria Bertani (LB; BBL, Becton Dickinson, Sparks, MD) agar for *E. tracheiphila* and *S. enterica*, respectively, after ten successive transfers in LB broth. Both pathogens were stored in LB broth aliquots, amended with 25% glycerol, at −80°C. For use in experiments, *S. enterica* and *E. tracheiphila* were grown on LB agar amended with gentamycin (30 µg/ml) (LB_gent._), and nutrient agar amended with ampicillin (100 µg/ml - NAP_amp._), at 37°C and 28°C, respectively, for 48 h. Bacterial cells were harvested with a sterile plastic loop and dispersed well in 0.1% peptone water to a final homogenous suspension of ca. 2×10^7^ cfu/ml, determined by optical density (OD) at 600 nm. To prepare mixed strain inoculum, equal volumes of each bacterial suspension were mixed to yield a final concentration of ca. 10^7^ cfu/ml. The inoculum titer was determined by plating appropriate dilutions (in 0.1% peptone water) on agar plates.

### Plant management

Seeds of cantaloupe, cv. Sugarcube, were sown 2.5 cm deep in cells of polypropylene flats containing Redi-earth potting mix (SUNGRO, Bellevue, WA) and placed in a growth chamber (24°C, 60% humidity,14 h day/10 h night). Each seedling (21 days old, 2–3 leaf stage) was transplanted to an individual pot (15.2 L capacity) containing Metromix-300 potting mix (Sun Gro, WA) supplemented with slow-release Osmocote fertilizer (19N, 6P and 12 K). Pots were transferred to the greenhouse, where the temperature was set at 24°C (day) and 18°C (night) with 14 h day/10 h night periods; humidity averaged 52% throughout the experiment.

A week after transplanting, vines were trained vertically and tied onto a framework of polyvinyl chloride (PVC) pipes to minimize plant-to-plant contact and to facilitate sampling from individual plants. Pots were watered every other day. Pistillate flowers were pollinated, using a fine artist's paint brush, with pollen collected from 1–2 staminate flowers of the same plant. Resulting young fruit were attached to the PVC frame so that, after inoculation, they were free from contact with other plant parts or the PVC frame.

### Experimental design

The cantaloupe plants were divided into four groups, each of which received one of 4 treatments: *E. tracheiphila* only (*Et*), *S. enterica* Poona only (*S*P), a mixture of these two pathogens (*Et*+*S*P), or 0.1% peptone water. Each cantaloupe plant was allowed to produce either two or three fruits (depending on the sampling time, see below). Fruits were harvested at 0, 9 or 24 days post inoculation (DPI). For experimental convenience, plants were assigned into two categories; i.e. in each replication, for each pathogen inoculation treatment (Et, SP, and Et+SP), 3 plants (group 1) were allowed to produce 3 fruits/plant (one to be sampled at 0 DPI, one at 9 DPI, and one for bacterial translocation assessment at 24 DPI), and 5 plants (group 2) were allowed to produce 2 fruits/plant (one for sampling at 24 DPI and one for bacterial translocation assessment). In all treatments, the fruits designated for bacterial translocation assessment were not inoculated with any pathogens; they were harvested at the end of each experiment and their central mesocarps were assayed individually for S. *enterica* and *E. tracheiphila* as described below. As controls, for each replication, 1 plant (3 fruits/plant, one for 0 DPI, one for 9 DPI sampling and one for bacterial translocation assessment) and 2 plants (having 2 fruits each plant one to be sampled at 24 DPI and one for bacterial translocation assessment) were inoculated with sterile 0.1% peptone water as described below (total control plants = 9 and total fruits inoculated with peptone water = 12, the fruits designated for assessing bacterial translocation were not inoculated).

In each replication, plants of each treatment group were arranged in separate areas in the greenhouse to prevent potential contamination from touching. Although in each treatment, the fruits sampled at 0 DPI and 9 DPI were from the same plant (due to space limitation in the greenhouse), a minimum complication was predicted since the 0 DPI fruits were harvested immediately after inoculation leaving the plant to produce another suitable fruit for 9 DPI sampling. Therefore, the experimental unit for treatment and DPI is the fruit and the experiment was considered a completely randomized design.

### Inoculation of fruit rind

**Figure 1:**
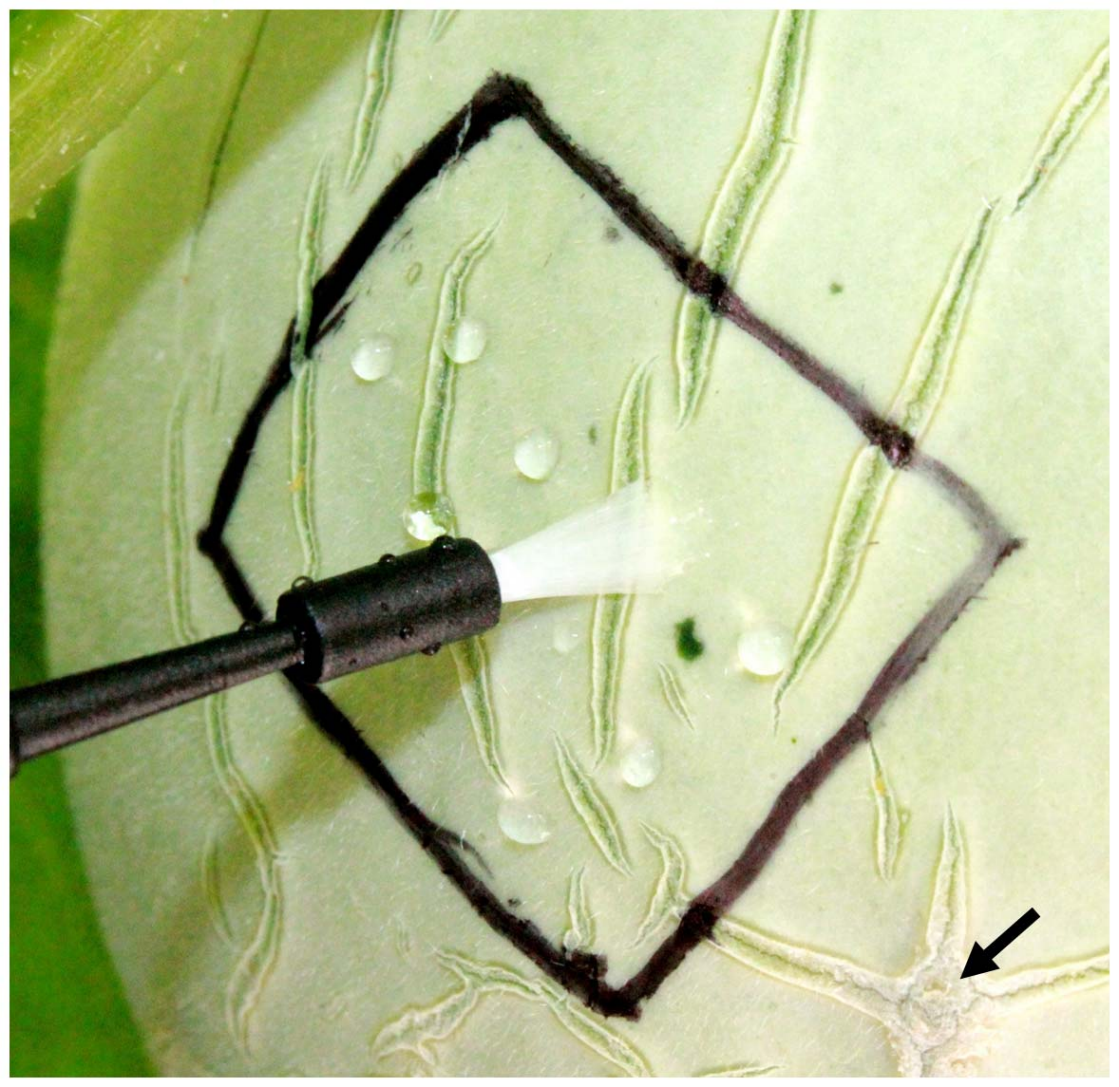
Inoculating newly formed natural cracks on cantaloupe fruit. Fruit rind (2×2 cm areas marked) was inoculated with pathogen suspensions or 0.1% peptone (20 µl as 10–15 droplets) and the droplets were then spread out in the marked area with a sterile bristled brush. Arrow head indicates cracks older than those in the area being inoculated.

Twelve-day-old fruits having fresh natural cracks were inoculated. Suspensions (20 µl) of *S. enterica*, *E. tracheiphila*, a mixture of both bacteria (ca. 10^7^ CFU/ml), or 0.1% peptone water, were deposited in 10–15 droplets onto the rind within a 2×2 cm square drawn with an indelible marker around a freshly formed crack on a single fruit of each plant (Fig. 1). The droplets were spread over the marked area including the crack using a new soft, sterile artist's brush for each inoculation.

### Fruit sampling and microbiological analysis

Fruit were sampled immediately after inoculation (0 DPI), at 9 DPI and at fruit maturity (when fruits easily detached from peduncles, averaged as 24 DPI). Fruit sampled at 9 DPI and at maturity were examined for symptom development and any change in the appearance of the inoculation site was noted. The marked squares were slightly larger at these sampling dates than at the time of inoculation because of fruit expansion. Samples from inoculated fruit consisted of fruit rind pieces (2×2 cm^2^, 2–3 mm thick, from within the inoculated square) and fragments of the sub-rind mesocarp excised from immediately beneath the inoculation site (∼2 cm thick, 7–10 g each). From un-inoculated fruit on the same plants, which were left in place to test for possible systemic bacterial spread, only the mesocarp (∼25 g, including seeds) from the center interior was sampled for pathogen presence. Surface rind and sub-rind mesocarp samples were excised aseptically from the inoculated 2×2 cm squares, whereas central mesocarp samples were excised from the centers of whole un-inoculated fruit. The rind fragment was divided into two unequal parts; one (3 cm^2^ and 2–3 mm thick) was used for microbiological analysis (cultivation and enumeration of viable microbes and PCR) and the other (1 cm^2^) was processed for analysis with confocal laser scanning microscopy (CLSM) and scanning electron microscopy (SEM). If watersoaking was present, the 1 cm^2^ sample was collected from the watersoaked area; otherwise the sample was excised from one corner of the 2×2 cm rind piece.

Rind pieces (3 cm^2^×2–3 mm thick) were placed into sterile whirl-pak bags (7 oz., Nasco, WI) containing 10 ml Universal Pre-enrichment Broth (UPB) (Becton, Dickinson, Sparks, MD) and hand massaged from the outside with firm pressure for 2 min followed by 1 min of vigorous hand shaking. Samples of sub-rind mesocarp and central mesocarp were placed separately in whirl-pak bags having filters (24 oz. and 55 oz. capacity, respectively) and macerated with a rubber hammer. UPB was added at a ratio of 1∶9 (wt∶vol). A 100 µl volume of each of these homogenates was plated (two replicates) on NA supplemented with ampicillin (NA_amp_, 100 µg/ml) and xylose lysine deoxycholate (XLD) medium (Becton Dickinson, Sparks, MD) for enumeration of microbes present at high titers, and 250 µl volumes of the same aliquots were plated on each of 4 XLD and 4 NAP_amp._ plates for enumeration of microbes present at low titers. XLD plates, selective for *Salmonella*, were incubated at 37°C for 24 h, and NAP_amp_, selective for GFPuv tagged *E. tracheiphila*, were incubated at 28°C for 3–4 days. The remaining suspensions were incubated at 28°C for 24 h, and then loopfuls of the enriched UPB were streaked onto XLD and NAP_amp_ plates and incubated as above. To enrich selectively for *S. enterica*, 100 µl of the overnight enrichment culture was transferred to 10 ml of Rappaport Vasilliadis broth (RV) (Becton, Dickinson, Sparks, MD) and incubated at 42°C for 48 hrs. A loopful of incubated RV broth was streaked onto XLD plates and incubated for 18–24 h at 37°C to observe black colonies that were presumptive of *Salmonella* Poona.

### PCR confirmation of *S. enterica* and *E. tracheiphila*

Aliquots (1 ml) of overnight incubated rind and mesocarp samples were centrifuged (5800× *g*, 10 min) and the pellets stored at −20°C until the DNA was extracted for PCR. DNA was extracted using a DNeasy Blood and Tissue Kit (QIAGEN, Austin, TX). Pathogen presence was assessed by a multiplex PCR using *Salmonella* specific primers (forward- 5′- GTGAAATTATCGCCACGTTCGGGCAA-3′ and reverse- 5′- TCA TCGCACCGTCAAAGGAACC-3′) to amplify a 284-bp nucleotide sequence within the *invA* gene [39] and *E. tracheiphila* specific primers ETC1 (5′-GCACCAATTCCGCAGTCAAG-3′) and ETC2 (5′-CGCAGGATGTTACGCTTAACG-3′) to amplify a 426-bp nucleotide sequence within the carbamoylphosphate synthetase gene [40]. PCR was carried out in a 25 µl reaction consisting of 12 µl Gotaq Green Mastermix (Promega, Madison, WI), 3 µl template DNA, 1 µl of each primer (total 4 µl), and 6 µl of nuclease free water. PCR was performed in an Eppendorf thermal cycler (Eppendorf, Hauppauge, NY) with cycling conditions including an initial denaturation at 94°C for 3 min, followed by 35 cycles at 94°C for 30 s, 60°C for 20 s, 72°C for 30 s, and a final extension at 72°C for 3 min. Amplified products were electrophoresed (90 volts) on 1.5% agarose gel made with 1× TAE buffer for 1 h. The experiment was replicated three times.

### Confocal laser scanning microscopy (CLSM)

To locate the inoculated pathogens on the fruit rind, a 1 cm^2^ rind piece from the 4 cm^2^ inoculated square was divided into two parts (0.5×1×0.2–0.3 cm each) for analysis by SEM and CLSM. A total of 36 samples (3 DPI×4 treatments×3 replications) were processed for both types of microscopy. Tissues were fixed in 4% paraformaldehyde for 1 h and washed 3× in distilled water. Fixed pieces layered with a razor blade to yield three sections (0.5 cm^2^×1 mm or less thick) were mounted onto a glass slide. To visualize green (GFPuv) or red (DsRed) fluorescence - expressing *Erwinia* and *Salmonella*, respectively, sections were scanned using a LEICA (Japan) TCS SP2 Laser Scanning Confocal Microscope with an upright Leica DMRE microscope, equipped with an Argon ion laser at 458, 476, 488 and 514 nm; green HeNe at 543 nm; and red HeNe at 633 nm; the Coherent UV Laser was at 300–360 nm. GFPuv was found to excite with 488 nm light and the emission was collected through a BA 505–525 filter. The wavelength of the lasers was first optimized using positive control samples inoculated with both pathogens before processing the experimental samples.

### Scanning electron microscopy (SEM)

Each of the 36 remaining 0.5×1×0.2–0.3 cm rind pieces of fruit sampled at 0, 9, or 24 DPI was cut into quarters (totaling 144 pieces, each 0.25×0.5×0.2–0.3 cm) that were processed for SEM. Pieces were fixed with 2% gluteraldehyde in 0.2 M cacodylate buffer and stored at room temperature for 2 h, rinsed 3× with 0.1 M buffered wash (60 ml 0.2 M cacodylate buffer and 12.3 g sucrose dissolved in 140 ml of distilled water) and then fixed for 1 h in 1% osmium tetroxide at room temperature. After another rinse they were dehydrated in ethanol [(30%, 50%, 70%, 80%, 90%, 95%, and 100% (3 X)] followed by critical-point drying 2× with HMDS (hexamethyldisilazane) and sputter coating with Au/Pd for 2 min with a MED 010 sputtering device (Balzers Union, Blazers, Liechtenstein). Coated samples were examined at different magnifications with a Quanta 600F scanning electron microscope (FEI Corporation, Hillsboro, Oregon), operating at 15 to 20 kV.

### Statistical analysis

The experiment was performed in three replicates, conducted independently at different times, with a total of 30 plants per replicate (27 plants for all treatments plus 3 as backups). Mean and standard errors of log base 10 transformed colony counts of both bacteria were calculated using MS Excel and the resulting data were analyzed using analysis of variance procedures with SAS Version 9.2 (PROC MIXED, SAS Institute, Cary, NC). A two-factor factorial model in a completely randomized design was utilized with the factors of interest being treatment and DPI. Simple effect means for DPI given treatment and treatment given DPI were reported and analyzed with planned contrasts. To avoid complications of heterogeneity of variance, treatment combinations that resulted in colony counts of zero log were excluded from the analysis and comparisons to those means were made with univariate t-tests. Data representing the percent of fruit that had *Et* lesions or that were positive for *S*P at different sampling times were analyzed with contingency tables and Fisher's Exact Tests. All statistical tests are considered significant at the P≤0.05.

## Results

### Fruit appearance and symptom development

**Figure 2:**
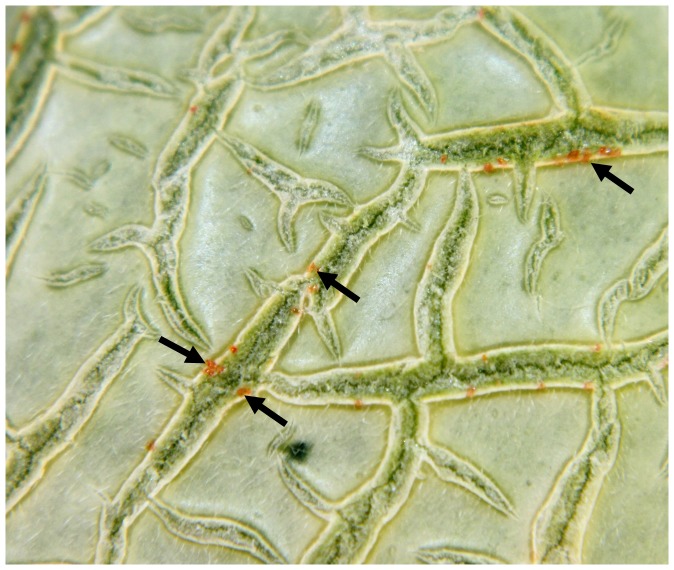
Reddish-orange exudate (arrow) observed on the natural cracks of 10–12 day-old cantaloupe fruit rind. These cracks were naturally healed by deposition of corky material, forming the characteristic netting on cantaloupes at fruit maturity.

Newly formed, healthy cantaloupe fruit were smooth-skinned with fine hairs, but at about 10–12 days of age small cracks appeared in the rind near the blossom end. Red-to-orange exudates seeping from the newly formed rind cracks (Fig. 2) indicated the presence of a connection from the fruit interior to the outside environment. The cracks lengthened, branched and intersected over time, gradually filled in and became raised as corky layers built up along them.

**Figure 3:**
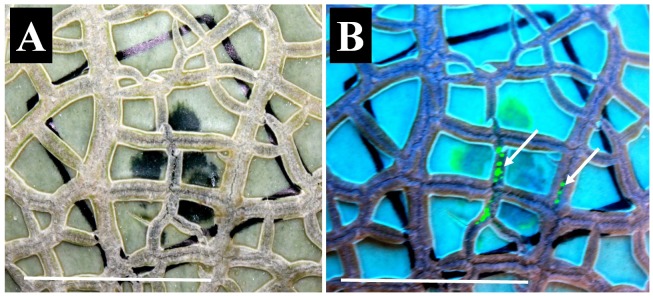
Watersoaked lesion observed under natural light (A) and UV light (B). Cantaloupe rinds were inoculated with *E. tracheiphila* (*Et-* green fluorescing with GFPuv), alone or together with *S. enterica* (*S*P- red fluorescing with DsRedExpress), and sampled at 0, 9 or 24 DPI. This rind, sampled at 9 DPI, showed watersoaked lesions observed under natural light (A) and under UV light (B). Arrows: Green-fluorescing *E. tracheiphila* on the cracks and beneath the cuticle in a watersoaked area. Scale bars represent 2 cm.

**Figure 4:**
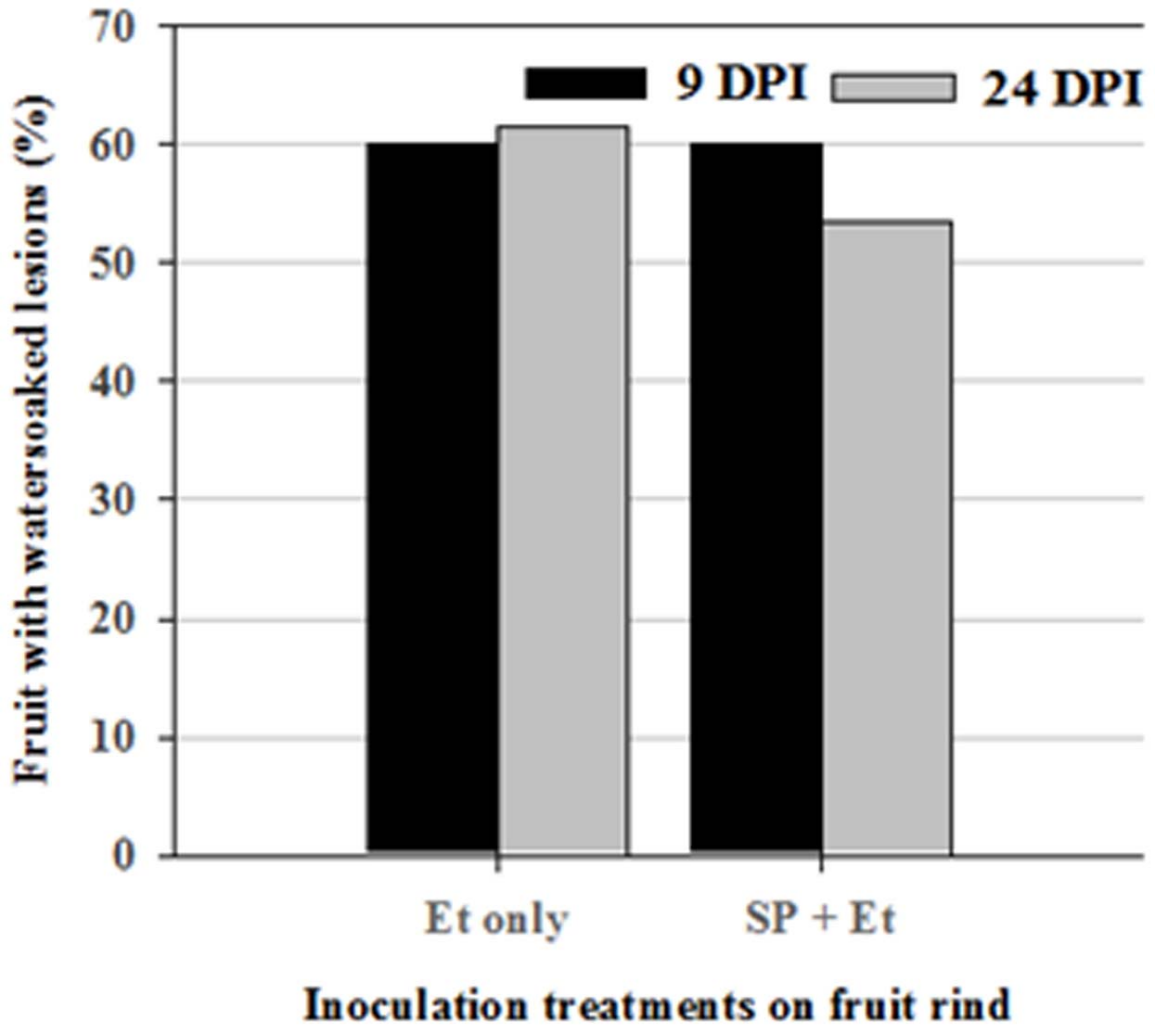
Disease incidence on cantaloupe fruits following inoculation with pathogens. Fruit rinds were inoculated with *E. tracheiphila* or a mixture of *E. tracheiphila* and *S. enterica* and sampled at 9 and 24 DPI. Bars indicate percentages of cantaloupe fruit showing watersoaking; there were no significant differences between day 9 and 24 for either treatment (*Et* or *S*P+*Et*) (Fisher's exact one-tailed test, P = 0.64 and P = 0.53, respectively).

After *E. tracheiphila* or *E. tracheiphila + S. enterica* inoculation of cantaloupe fruit rind, small watersoaked lesions appeared at the inoculated site within 4–7 days on 28 (58%) of the inoculated fruit. *E. tracheiphila*, tagged with GFPuv, was observed (using UV light) as patches of green fluorescence between the rind cracks and underneath the rind cuticle; over time the fluorescence spread from the crack into the underlying tissues, suggesting that the crack was the point of *E. tracheiphila* entry (Fig. 3 A and B). No lesions appeared on any fruit inoculated with *S. enterica* alone or the control buffer. Most (61%) of the fruit inoculated with *E. tracheiphila-*alone and sampled at 24 DPI, had watersoaked lesions that ranged from barely noticeable to a maximum of ca. 2 cm^2^, reaching as large as half the area of the inoculation spot on a few fruits. The percentage of fruit that developed watersoaked lesions by 9 and 24 DPI in these two treatments did not differ significantly (P<0.05) (Fig. 4).

### *S. enterica* and *E. tracheiphila* survival on cantaloupe fruit rind

After inoculation of *S. enterica*, *E. tracheiphila*, or a mixture of both species onto cantaloupe fruit rind, bacterial recovery varied with the sampling time. Recoveries of *S. enterica* by direct plating at 0 DPI were 3.62 and 3.69 log out of 5.60 log CFU/3 cm^2^ inoculated bacteria in the *S. enterica*-only and the *S. enterica* + *E. tracheiphila* inoculated treatments, respectively (Table 1). *S. enterica* numbers recovered in both treatments were significantly (p<0.0001) lower at 9 DPI than at 0 DPI, and by 24 DPI only 2 fruit (13%) receiving the *S. enterica + E. tracheiphila* treatment still had numbers of *S. enterica* detectable by direct plating (Table 1).

**Table 1:** Recovery of *S. enterica* Poona and *E. tracheiphila* from cantaloupe rinds.

Treatment^1^	Pathogen assessed	Presence of watersoaked lesion	Pathogen recovery^2^ (Log CFU/3 cm^2^)		
			0 DPI	9 DPI	24 DPI
*Et*	*Et*	+	NA^3^	TNTC^4^ (6/10)	TNTC (8/13)
		−	1.58^a^±0.30 (8/10)	0.00^b^±0.00 (4/10)	0.00^b^±0.00 (5/13)
*S*P	*S*P	−	3.62^Aa^±0.19 (9/9)	0.65^Ab^±0.27 (4/9)	0.00^Ac^±0.00 (0/14)
*S*P+*Et*	*Et*	+	NA	TNTC (6/10)	TNTC (8/15)
		−	0.49^a^±0.34 (2/10)	0.00^b^±0.00 (4/10)	0.00^b^±0.00 (0/15)
	*S*P	+	NA	0.82^Aa^±0.39 (6/10)	0.24^Aa^±0.24 (8/15)
		−	3.69^Aa^±0.19 (10/10)	0.75^Ab^±0.47 (4/10)	0.31^Ab^±0.31 (7/15)
Control	*S*P+*Et*	−	0.00±0.00 (0/3)	0.00±0.00 (0/3)	0.00±0.00 (0/6)

1 Et – *Erwinia tracheiphila*; SP – *Salmonella enterica* Poona.

2 Cantaloupe rind pieces (3 cm^2^×2–3 mm deep) were sampled at 0, 9, or 24 days post inoculation (DPI); values are means ± standard deviation of the means; numbers in parenthesis following pathogen recovery values indicate # of fruits having detectable level of pathogens (by direct plating)/total # fruits sampled, the detection limit by direct plating is l log CFU/3 cm^2^; means within the same row having the same lowercase letters or within the same column having the same capital letters are not statistically significant at the 0.05 level.

3 NA- Not applicable as none of the rind surfaces having lesions yet right after pathogen inoculation (0 DPI).

4 TNTC-too numerous to count.

Unlike with *S. enterica*, *E. tracheiphila* recovery by direct plating was very low even at 0 DPI. Only 1.58 log CFU/3 cm^2^ and 0.49 log CFU/3 cm^2^ were recovered from fruits treated with *E. tracheiphila* alone, or with *E. tracheiphila* + *S. enterica*, respectively, out of 5.60–5.70 log CFU/3 cm^2^ inoculated (Table 1). The latter recovery rate was ca. 70% lower than that of the *E. tracheiphila*-only treatment. *E. tracheiphila* was not detected on fruits sampled at 9 and 24 DPI in either treatment when watersoaked lesions were not present.

### Microscopy

**Figure 5:**
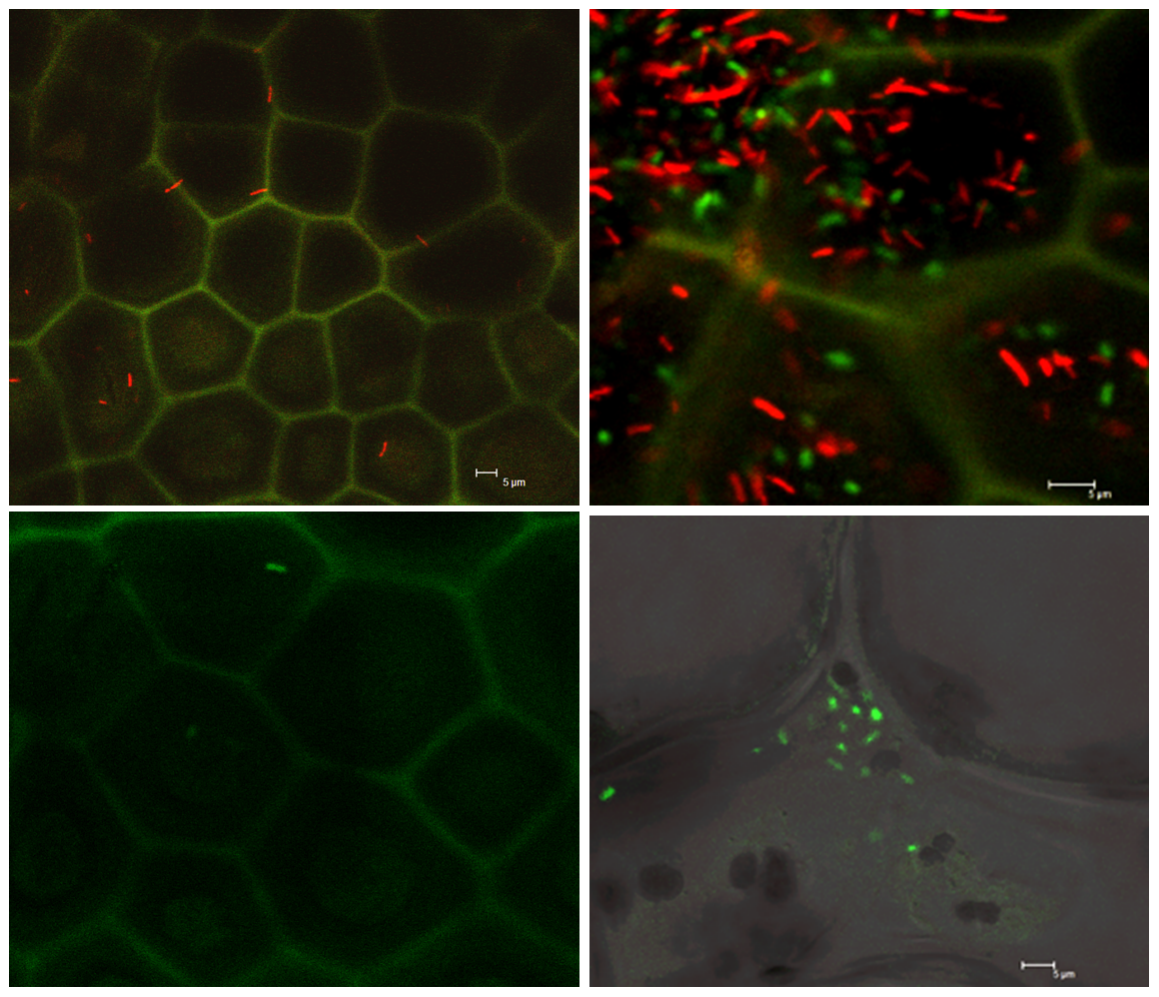
Confocal laser scanning microscope images of cantaloupe rind surfaces. (A) Fruit rind surface inoculated with *S. enterica* Poona (labeled with DsRedExpress) and sampled at 0 DPI, (B) Fruit rind surface inoculated with a mixture of *S. enterica* Poona + *E. tracheiphila* (labeled with GFPuv) and sampled at 0 DPI (C) Fruit rind surface inoculated with *E. tracheiphila* and sampled at 0 DPI and (D) Longitudinal section of rind containing watersoaked lesion and sampled at 24 DPI; *E. tracheiphila* in the intercellular spaces (arrow) (inoculated with mixture of *S. enterica* plus *E. tracheiphila*). The scale bars represent 5 µm.

At 0 DPI *S. enterica* was observed by CLSM on rind samples that had received both single and mixed culture inoculations (Fig. 5 A and B, respectively). Interestingly, we observed irregularly spaced clusters of red cells (*Salmonella*) on the same sample pieces, suggesting an uneven distribution of the inoculated cells on the rind surface. Only a few samples were visually positive (on the surface) for *S. enterica* at 9 DPI (2 out of 6 samples inspected), and none were visually positive at 24 DPI (data not shown). *E. tracheiphila* was observed on the rind surface at 0 DPI (Fig. 5 C), and at 9 and 24 DPI when watersoaked lesions were present (Table 2). In internal layered sections below the watersoaked lesions (to a depth of 3 mm), *E. tracheiphila* was observed in the intercellular spaces at 9 and 24 DPI (Fig. 5 D).

**Table 2:** Association, based on PCR detection, of *E. tracheiphila*-incited watersoaked lesions with the presence of *S. enterica* on cantaloupe fruits sampled over time*.

Days post-inoculation	*S. enterica-*positive fruits with lesions (%)	*S. enterica-*positive fruits without lesion (%)	P- value
9	83.3 (5/6)*	75.0 (3/4)	0.67
24	50.0 (4/8)	28.5 (2/7)	0.38
Total	64.3 (9/14)	45.5 (5/11)	0.30

*Comparison, by Fisher's Exact Test, of percent of fruit PCR positive for *S. enterica* on cantaloupe fruit having *E. tracheiphila* induced lesions vs. fruits without lesions.

**Figure 6:**
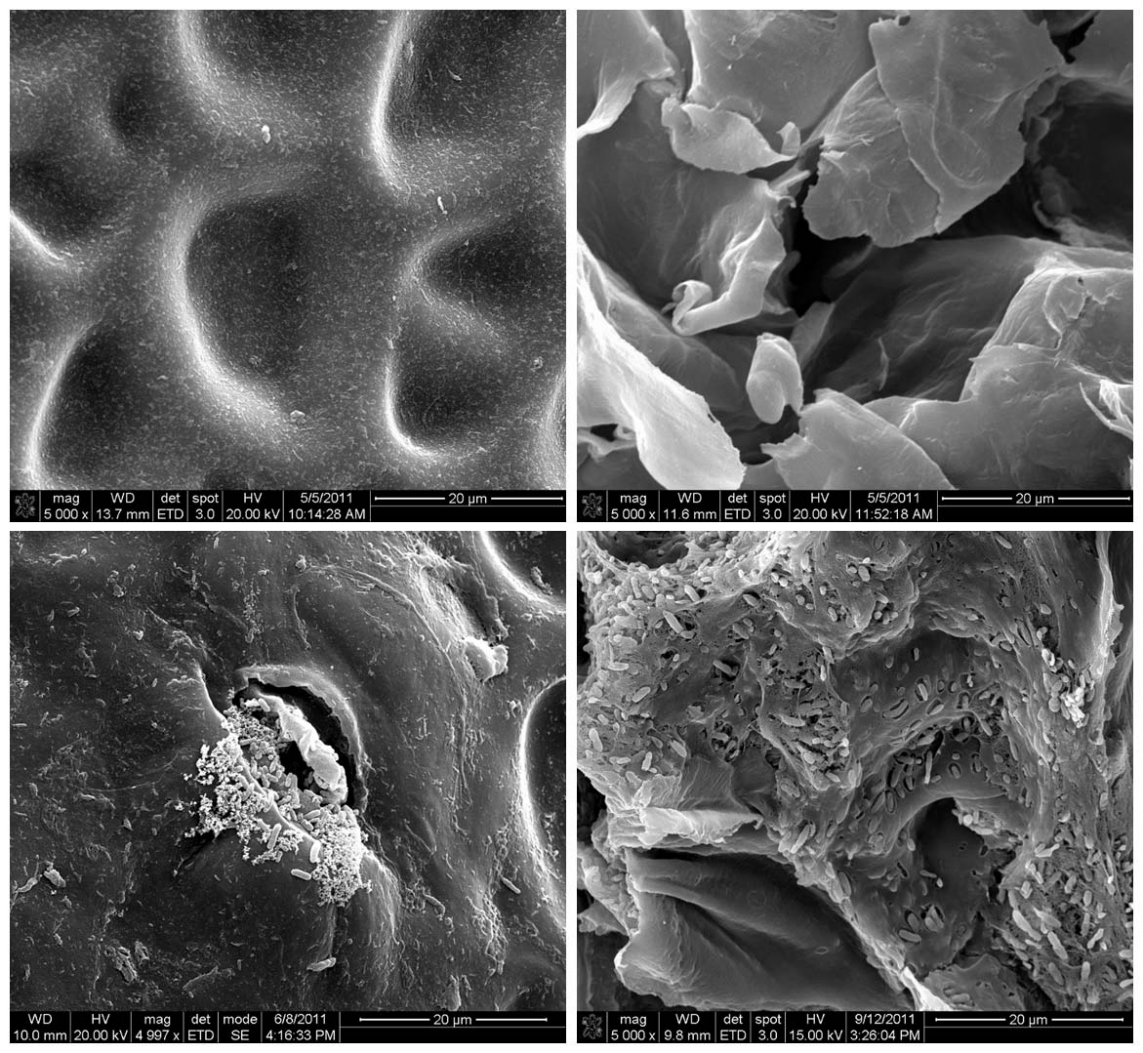
Scanning electron micrographs of cantaloupe rind surface at fruit maturity. (A) Rind inoculated with 0.1% peptone water; (B) Crack on rind inoculated with 0.1% peptone water; (C) Rind inoculated with *E. tracheiphila* and had a watersoaked lesion with masses of bacteria seen near a trichome scar; and (D) Crack on rind inoculated with mixed *S. enterica* + *E. tracheiphila* that had a waterloaked lesion. All observations were made at 5,000×; scale bar shows 20 µm.

Scanning electron micrographs of the rind surface (Fig. 6 A) and cracks (Fig. 6B) showed that fruit inoculated with *E. tracheiphila* or *E. tracheiphila* + *S. enterica*, having watersoaked lesions, harbored bacterial masses on the rind surface on or adjacent to the natural cracks (Fig. 6 C) as well as deep inside the cracks (Fig. 6 D). Bacteria-inoculated fruits lacking lesions and control fruits had no visible bacteria.

### Detection by PCR and enrichment culture

**Figure 7:**
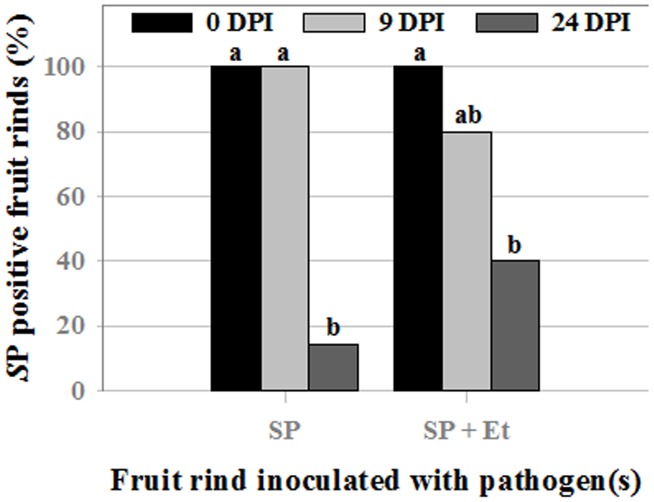
Incidence of *S. enterica* Poona (*S*P) on cantaloupe fruits. Fruit rinds were inoculated with *S. enterica* Poona alone (*S*P), or *S. enterica* + *E. tracheiphila* (*S*P+*Et*), and sampled at 0, 9 or 24 days post inoculation [(DPI), P values for *S*P vs *S.*P+*Et* at 0, 9 and 24 DPI were 1.00, 0.47, and 0.21]. Values of the same treatment do not significantly differ at p<0.05) according Fischer's Exact test- one tailed. Overall p-value for comparison of proportions among levels of DPI given treatments are <0.001 and 0.0039 for *S*P and *S*P+*Et*, respectively.

The number of *S. enterica* positive fruits, as determined both by PCR and enrichment culture, were significantly higher (P>0.001) at 0 DPI than at 24 DPI in both single and multispecies inoculated samples (Table 1, Fig. 7). *S. enterica* was detected (by overnight enrichment culture and PCR) on 14% and 40% of fruit inoculated with *S. enterica*, or with *S. enterica* + *E. tracheiphila*, respectively, at 24 DPI, but these treatment differences were not significant (P = 0.11, one tailed Fisher's Exact Test) (Table 1, Fig. 7). In all cases, fruits positive by PCR were positive also by enrichment culture, and vice versa. Among fruit inoculated with mixed culture and sampled at 24 DPI, *S. enterica* was detected on more fruits (50% - 4 out of 8) having *E. tracheiphila -* induced watersoaked lesions than on those without them (28.5% - 2 out of 7), although these differences were not significantly different either (P = 0.6084) (Table 2).

### *S. enterica* colonization of cantaloupe fruit mesocarp

Two types of mesocarp samples, one immediately underneath the *S. enterica* or *S. enterica* + *E. tracheiphila* inoculated rind and sampled at 0, 9 and 24 DPI (i.e. sub-rind mesocarp) and the other from the central core of fruits that received no rind inoculations and were sampled only at 24 DPI (i.e. central mesocarp), were examined. Neither microbial analysis (cultivation) nor PCR ever revealed *S. enterica* in the sub-rind mesocarp of 112 fruits sampled in all DPI and treatments (data not shown).

### Assessment of bacterial systemic movement

Eighty one inner mesocarp samples were taken at 24 DPI from the central core of fruits that received no inoculation but were growing on the same plants on which other fruits received either 0.1% peptone water, *E. tracheiphila*, *S. enterica*, or *S. enterica* + *E. tracheiphila*. All of the inner mesocarp samples were negative for *S. enterica* by both microbial plating and PCR (data not shown).

### *E. tracheiphila* colonization of cantaloupe fruit sub-rind mesocarp

Some of the sub-rind mesocarp samples of fruit that were inoculated with *E. tracheiphila* or *E. tracheiphila* + *S. enterica*, and that later developed watersoaked lesions (sampled at 9 DPI and later), were positive for *E. tracheiphila* by microscopy, culture and PCR. On fruit inoculated with *E. tracheiphila* only, this bacterium was detected in one out of ten (10%) fruits and in 4 out of 13 fruits (31%) of sub-rind mesocarps sampled at 9 and 24 DPI, respectively. At 24 DPI, 27% of the sub-rind samples that received *E. tracheiphila* + *S. enterica* and had watersoaked lesions were positive for *E. tracheiphila*. All control fruit and all fruit that did not develop watersoaked lesions were negative for both pathogens on sampled sub-rind mesocarp.

## Discussion

Repeated outbreaks of foodborne illness associated with *S. enterica* contaminated cantaloupe demonstrate the importance of understanding the mechanisms of microbial contamination and persistence in this fruit. Our knowledge of factors that influence the fate of enteric human pathogens on plant surfaces is expanding with recent research providing evidence that other species in the microbial phylloplane community, including plant pathogens, can influence not only the behavior and persistence of human pathogens on plant surfaces but also their propensity to enter plant interiors, and even to manipulate plant systems such as stomatal control and defense strategies [41]–[43]. In this study we investigated the fate of *S. enterica* Poona, alone or in the presence of the cucurbit wilt causing bacterium, *E. tracheiphila*, on cantaloupe fruit surfaces.

An unusual feature of developing cantaloupe fruit is the formation, during fruit expansion, of an extensive network of cracks that penetrate through the rind [16],[44], and the subsequent deposition of a corky seal containing lenticels that becomes the netting that characterizes the rind of cantaloupes [16],[45]. Prior to the wound healing, however, the cracks may provide a pathway for microbes on the rind surface to enter interior tissues.

In our work, after inoculation, alone or in a mixture with *E. tracheiphila*, *S. enterica* could be detected on cantaloupe rind surfaces throughout the duration of the experiment, but its population levels declined over time (sampling at 0, 9 and 24 DPI) irrespective of the treatment. Our results are consistent with the findings of others who reported similar decline of *S. enterica* numbers over time after inoculation on produce [24],[46]–[48]. This finding is not surprising, as many factors influence bacterial survival and the plant environment is generally not considered as a natural niche for human enteric pathogens [24],[46]–[48]. Although most fruits receiving *S. enterica* in our experiments tested positive by microbial cultivation only after enrichment, two fruits inoculated with the *S. enterica + E. tracheiphila* mixture, and sampled at 24 DPI, retained levels of *S. enterica* detectable through direct plating. Others also have shown that *S. enterica* can remain viable on some hosts (*Arabidopsis thaliana*, lettuce, parsley, radish, and carrot phyllospheres) for extended periods [49]–[51].

We found no evidence for invasion of the edible fruit mesocarp (sub-rind or inner mesocarp) by *S. enterica*, even when it was inoculated together with the plant pathogen *E. tracheiphila* and the latter had induced watersoaked lesions on the rind. Human enteric pathogens are documented plant invaders under some conditions, having been reported to traverse lettuce stomata [30] and roots [52],[53], as well as tomato stomata [27], hydathodes [29] and roots [26], flowers [54] and leaf trichomes [46]. The fact that we did not detect *S. enterica* in any mesocarp samples during our study suggests that this bacterium only rarely, if ever, traverses the rind into the edible portions of the fruit.

*E. tracheiphila*, which causes wilt in cantaloupe and many other cucurbit crops in the eastern United States [40],[55]–[57], is transmitted in nature by spotted and striped cucumber beetles [58],[59] as they feed on flowers, releasing bacteria that then infect the plant. Introduction of *E. tracheiphila*, in volumes and titers higher than those natural in the environment, directly onto cantaloupe rind surfaces is far from a natural phenomenon. However, our preliminary experiments had revealed that *E. tracheiphila* could enter the fruit after introduction to the cracked areas (data not shown) or through flower interiors [60], and that watersoaked lesions often appeared within 4–5 days or 18 days after inoculation, respectively. Furthermore, Rojas and Gleason [61] recently reported that *E. tracheiphila* can live as an epiphyte on muskmelon leaves under a wide range of leaf wetness levels and temperatures, and they speculated that this niche could serve as a source of *E. tracheiphila* inoculum for pathogen dissemination. Their findings, combined with ours, suggest that *E. tracheiphila* may be a common resident on cucurbit plant surfaces in nature. If this is true, then its ability to facilitate the survival of a human pathogen such as *S. enterica* becomes much more than an academic issue.

*E. tracheiphila* was detected by culture plating on fruit rind soon after inoculation at 0 DPI, but at numbers significantly lower than expected based on the amount inoculated, and it was never detected by culturing from the surfaces of healthy looking fruit (fruits without watersoaked lesions) at 9 and 24 DPI. However, confocal microscopy of the same samples revealed green fluorescent bacteria (*E. tracheiphila)* in numbers similar to those of *Salmonella* (red fluorescent) (Figure 5B), suggesting that direct plating of *E. tracheiphila*, which is somewhat fastidious in laboratory culturing [62],[63], might not accurately reflect actual numbers of the bacterium in a sample. Considerable research has been done to find the most appropriate inoculation [64]–[70], isolation [64],[67], and storage [58],[71] techniques for *E. tracheiphila*. Numerous methods of inoculating *E. tracheiphila* to cucurbit plants have been reported [72], but to our knowledge this is the first report of its ability to internalize through the expansion cracks on the fruit surface. In our experiment, *E. tracheiphila* did traverse the rind of some fruits (61%), leading to the formation of watersoaked lesions that enlarged over time. We detected *E. tracheiphila* in 31% of sub-rind mesocarp samples from fruits that had received the *E. tracheiphila* alone treatment, and that had developed watersoaked lesions at 24 DPI. These bacteria increased in number in that location from 9 DPI to 24 DPI, suggesting that they either continue to move there over time or multiply there. That *E. tracheiphila* can colonize the cantaloupe rind surface, enter the underlying mesocarp tissue through natural cracks, and cause watersoaked lesions is a new finding, but these phenomena occurred after bacteria were deposited artificially in high numbers on the fruit rind. However, it could be hypothesized that such events might take place in nature, but be un-noticed, if contaminated beetles feed on these fruits or their frass contaminates open cracks on the fruit surface.

Introducing the human pathogen, *S. enterica*, and the plant pathogen, *E. tracheiphila*, simultaneously led to some differences in the behavior of the individual bacterial species. In this work, although not statistically significant, possibly due to the relatively small number of fruits tested, a higher percentage of rinds were PCR-positive at fruit maturity (24 DPI) for *S. enterica* in the presence of watersoaked lesions caused by *E. tracheiphila* than on non-symptomatic rinds (Table 2). In nature, human pathogens that come into contact with potential plant niches encounter resident microflora with which they may interact synergistically or antagonistically [34],[73]–[75]. Klerks et al. [52] demonstrated an inverse relationship between the diversity of the endophytic microbial community and inoculated *Salmonella* survival in lettuce leaves and a similar observation reported by Gu et al. [28] on tomato leaves. Microbial synergism between *S. enterica* and normal plant microflora, such as certain storage [35] and pathogenic fungi [33], and the plant pathogen *Xanthomonas campestris* pv. *vesicatoria*, in the absence of plant disease [24], has been reported. Recently, Pontis et al. [32], showed a positive correlation between the presence of virulent *Xanthomonas perforans* and *S. enterica* survival on the tomato phyllosphere. Our test pathogens, i.e. *S. enterica* and *E. tracheiphila*, might interact and colonize differently on other varieties of cantaloupe fruit; a study with *S. enterica* and *Escherichia coli* O157:H7 showed variable levels of root colonization depending on the cantaloupe variety [76]. In the latter study, both *S. enterica* and *E. coli* colonized the rhizosphere of ‘Burpee's Ambrosia’ most and ‘Israel Old Original’ least among five cultivars tested. In the work reported here, we cannot conclude that *S. enterica* survival was enhanced by the presence of *E. tracheiphila*, although the leakage of cellular contents into intercellular spaces after *E. tracheiphila* inoculation, causing watersoaking, could provide nutrients and water supportive of *S. enterica* growth on the rind surface, thereby potentially affecting the persistence of the human pathogen in what would otherwise have been a less favorable environment.

We saw no indication that the presence of *S. enterica* influenced the behavior or survival of *E. trachiphila* on the cantaloupe fruit, an interesting observation since, in vitro, when *S. enterica* and *E. tracheiphila* were streaked together onto the same agar plate, there was clear inhibition of the latter by the former (data not shown).

In this work there was no evidence for systemic movement of either pathogen in the cantaloupe plant after rind inoculation. Lopez-Velasco et al. [17] showed that *Salmonella*, introduced into cantaloupe peduncles, was found in adjacent acropetal and basipetal tissues, suggesting the possibility of limited movement within the peduncle. More interesting is that *E. tracheiphila*, which we detected in the fruit mesocarp after inoculation to the rind surface, and which, in “typical” wilt disease, moves systemically in the xylem [40],[58],[64],[72], was not detected in un-inoculated fruit present on the same plants that had inoculated fruit. Did our strain of *E. tracheiphila* lack some factors required for xylem access or systemic movement? In a preliminary experiment [60] we found evidence for systemic movement of this *E. tracheiphila* strain, first to the adjacent fruit and subsequently to the vines, after inoculation into flower interiors, resulting in plant wilting. In the work reported here, however, the short (twenty four day) experimental period in this study may not have provided sufficient time for the plant pathogen to move into the vines and cause wilting. Furthermore, changes in fruit physiology or anatomy during ripening may restrict systemic bacterial spread, a hypothesis consistent with our finding that *E. tracheiphila-*incited watersoaked lesions eventually became necrotic and ceased to enlarge over time. Many storage and pathogenic fungi [77],[78] and a few bacteria (*Xanthomonas cucurbitae*, causing bacterial leaf spot, and *Acidovorax avenae* subsp. citrulli, causing bacterial fruit blotch) are active on mature fruit but factors influencing their tissue specificities have yet to be characterized [79].

Based on our findings, we cannot conclude that *S. enterica* survival was enhanced by the presence of *E. tracheiphila*, but reports by others have shown that survival of *S. enterica* on plants is influenced by interactions, including synergism, with other microflora including plant pathogens. To our knowledge, this is the first report of the capability of *E. tracheiphila*, a common cantaloupe resident and pathogen, to internalize through natural fruit cracks and produce watersoaked lesions. The fact that *S. enterica*, inoculated similarly, either by itself or together with *E. tracheiphila*, did not enter the plant in detectable numbers is consistent with results of other studies [80]–[84] in which human enteric pathogens on edible plants failed to internalize. However, others [27],[29] have reported that *S. enterica* does enter into plants, illustrating the complexity of interaction and internalization determinants. Since our study was done under consistent, controlled, BSL-2 compliant greenhouse conditions it may not reflect actual field conditions and the possibility of *S. enterica* internalization via fruit cracking cannot be completely ruled out. Furthermore, the close taxonomic relationship of these two members of the Enterobacteriaceae leaves open to possibility that, within the protected plant environment in natural settings, there may be horizontal gene transfer from *E. tracheiphila* to *S. enterica*, enabling the latter to adapt to the unique plant niche. These fascinating questions remain to be explored.
